# Initiative for the implementation of planetary health in postgraduate medical training and continuing medical education in Switzerland

**DOI:** 10.3205/zma001608

**Published:** 2023-05-15

**Authors:** Robin Rieser, Barbara Weil, Nadja Jenni, Monika Brodmann Maeder

**Affiliations:** 1 Swiss Medical Association FMH, Bern, Switzerland; 2 Swiss Institute for Medical Education SIME, Bern, Switzerland

**Keywords:** Planetary Health, climate change, continuing medical education, national strategy

## Abstract

The Swiss Medical Association FMH drew up the strategy “Planetary health – Strategy on the courses of action on climate change for the medical profession in Switzerland” in collaboration with the Swiss Institute for Medical Education SIME, the umbrella organisations and students. On 7 October 2021, the strategy was approved by the Swiss Medical Chamber with a budget of over CHF 380,000 (approx € 365,000). The first step in implementation involved setting up an advisory group which will tackle the concrete implementation of the strategy. This article provides an insight into the current state of work on the project with a focus on the measures in the areas of postgraduate medical training and continuing medical education. It is a work in progress.

## Abstract (de)

Die Verbindung der Schweizer Ärztinnen und Ärzte FMH hat die Strategie „Planetary Health – Strategie zu den Handlungsmöglichkeiten der Ärzteschaft in der Schweiz zum Klimawandel” in Zusammenarbeit mit dem Schweizerischen Institut für ärztliche Weiter- und Fortbildung SIWF, den Dachverbänden und den Studierenden verfasst. Am 7. Oktober 2021 wurde die Strategie von der Ärztekammer der FMH mit einem Budget über ca. 380'000 CHF (ca. 365'000 €) verabschiedet. Als erster Schritt zur Umsetzung wurde eine Begleitgruppe ins Leben gerufen, die nun die konkrete Umsetzung der Strategie in Angriff nehmen wird. Der vorliegende Artikel gibt einen Einblick in den aktuellen Stand der Projektarbeit mit dem Fokus auf Maßnahmen im Bereich der ärztlichen Weiter- und Fortbildung. Er soll als „work in progress“ verstanden werden.

## 1. Background

### 1.1. Planetary health and human health

The publication of the sixth Assessment Report of the Intergovernmental Panel on Climate Change (IPCC) [1] made it very clear that climate change could no longer be ignored. The impacts of climate change are many and varied: rising temperatures, melting glaciers, air pollution, biodiversity loss, extreme weather events and rising sea levels. These are just some of the observed impacts of climate change that influence individual and public health, as well as planetary health [2], [3], [4]. The anthropogenic change in the climate is leading to longer periods of drought and heatwaves, an increase in heavy precipitation and other extreme weather events. Chiefly responsible for this warming are the greenhouse gas emissions from human activities [1]. Owing to Switzerland’s geographic location at the heart of the European continent and relative proximity to the North Pole region which is seeing strong warming, and the potential feedback effects from the reduction in Alpine snow cover, Switzerland is particularly affected by climate change [5]. Since the mid-19th century, Switzerland’s climate has warmed by around 2°C. This temperature rise is nearly twice as high as the increase in the average global temperature [5]

The health sector also makes a significant contribution to domestic greenhouse gas emissions. According to the latest Lancet countdown report, internationally, healthcare sectors account for 4.6% of total emissions [6]. In the United States, for example, the level is particularly high, with the domestic healthcare sector accounting for 8-10% of total emissions [7], [8]. In the United Kingdom, that figure was 4-5% in 2017. Owing to the efforts of the National Health Service (NHS) [9] to become the world’s first net zero health system, this figure continues to fall [6]. The figures for Switzerland differ, depending on the study and calculation method. An international comparison of the carbon footprints of healthcare sectors by Pichler et al. [7] assumed a share of 5.9% for Switzerland in 2014. Meanwhile, Health Care without Harm put this figure at 6.7% for 2019 [10]. Climate change is a substantial threat to regional, global and planetary health and action is needed in various fields of activity and at various levels. 

In a public opinion poll conducted in Switzerland in 2022, the threat of climate change was considered people's second-biggest worry [https://www.gfsbern.ch/en/news/worry-barometer-2022/]. Youth movements such as “Fridays for Future” and “Climate strike” have attracted attention and raised awareness of the climate crisis. The younger generations vehemently and publicly support climate action and protection of the planet. These voices can also be found within the medical profession: medical students (Swiss Medical Students’ Association, swimsa) worked with the VSAO (Association of Swiss Assistant Doctors and Chief Physicians) to present the “Manifesto for a healthy future“ to the Delegates Assembly of the Swiss Medical Association (FMH), which it signed at its meeting of 3 September 2020 [11]. The signatory organisations support the protection and promotion of public health, recognise climate change as a substantial threat to regional and global health, and call for a healthy and sustainable future (see attachment 1 ). To live up to its shared responsibility in terms of climate change, the FMH drew up the strategy “Planetary Health – strategy on the courses of action on climate change for the medical profession in Switzerland” with the umbrella organisations and students. The strategy was approved by the Swiss Medical Chamber on 7 October 2021 with a budget of more than around CHF 380,000 (approx € 365,000). 

The strategy comprises goals in four different action areas:

- **Education and awareness:** Educating and informing the medical profession and patients to raise awareness of the importance of the impact of climate change on health and to help people understand how they can take action.
- **Mitigation:** Highlighting and implementing concrete actions to reduce the environmental footprint of the medical profession with the aim of minimising the greenhouse gas emissions of the health sector.
- **Adaptation:** Developing measures to respond to unavoidable climate and environmental change so that particularly vulnerable people are better protected from climate change.
- **Leading by example: **Doctors should be aware of their responsibility and utilise the trust they enjoy to encourage patients to adopt healthy and climate-friendly behaviours. This will help realise the vision of a sustainably healthy and climate-resilient Swiss health system. For each action area, goals have been drawn up which allow progress to be documented (see table 1 (Tab. 1)). An advisory group made up of representatives of the umbrella organisations affiliated with the FMH is responsible for prioritising the individual action areas.

“Planetary health” deals with the health of human civilisation and the state of the natural systems on which it depends. It therefore describes a concept in which the health of the planet is of central importance as the basis of human health and all life [1]. Planetary health is an extension of public health and global health, where use of natural resources is considered in context alongside the impact of the whole system on health and on future generations. The concept harnesses synergies through a holistic approach in which, for example, protection, preservation and improvement of the natural environment also stabilises the climate, protects health and supports a sustainable economy. 

The following is intended to show how the actors involved are planning to implement the FMH’s Planetary Health Strategy in postgraduate medical training and continuing medical education. It also seeks to provide an overview of the anticipated impact of the measures. 

#### 1.2. The Swiss medical association FMH

The Swiss medical association (FMH) works to ensure that all patients in Switzerland have access to high-quality and affordable medical care. In the political decision-making process, it promotes balanced representation of the interests of its members and supports collaboration between the various actors in the Swiss health system.

#### 1.3. The Swiss Institute for Medical Education SIME

The Swiss Institute for Medical Education (SIME) is the competence centre for the medical profession, authorities and educational institutions regarding postgraduate medical training and continuing medical education in Switzerland. By bringing together all the key actors and organisations in this area as an autonomous body from the FMH, the SIME guarantees high-quality postgraduate training and continuing education for doctors in over 120 specialist fields. In particular, it is responsible for making decisions on revisions to the Specialist Training Regulations, the granting and annulment of specialist titles, and approving postgraduate training programmes devised or revised by the medical specialty societies. The SIME is solely responsible for carrying out editorial amendments or modifications to the overarching Specialist Training Regulations and Continuing Education Regulations and postgraduate training programmes, and approving new continuing education programmes and material revisions. Additionally, the SIME can deploy experts to examine specific questions, and accredit non-specialist events devoted to ethics, health economics and medico-actuarial science that serve to improve patient safety, risk and error management, or training of emergency services.

## 2. Implementation of the FMH’s planetary health strategy in postgraduate training and continuing medical education

The FMH's Planetary Health Strategy and the goals it sets out are implemented in four areas: awareness and education; mitigation; adaptation; and leading by example (see above). 

The following seeks to highlight how planetary health could be thematically integrated in postgraduate medical training and continuing medical education. The initiatives are geared to the AMEE Consensus statement, which defines planetary health and education for sustainable healthcare as follows: 

*“… We define education for sustainable healthcare as the process of equipping current and future health professionals with the knowledge, values, confidence and capacity to provide environmentally sustainable services through health professions education… “ *[12]

This makes it clear that simply imparting knowledge is not enough to change doctors’ behaviour, or to make doctors in Switzerland into role models for their patients. Therefore, besides curricular changes, incentives are needed to encourage existing and newly-acquired knowledge to be put into practice. Potential ways of integrating planetary health in curricula will be outlined and systems for incentivising inclusion of the content will be presented. Aspects of transformative education, which will be particularly key in the field of planetary health, will also be incorporated. Interprofessional, interdisciplinary and transnational networks will help translate sufficient existing expertise into action and activities. Initiatives such as the Planetary Health Education Framework can help support exchange and dialogue [13].

Undergraduate medical training is not covered in this summary as the SIME is not responsible for undergraduate training in human medicine. 

### 2.1. Planetary health in postgraduate medical training

Postgraduate training is the activity of the doctor after successfully completing medical studies with a view to acquiring a specialist’s title certifying their qualification to practise their medical activity in a chosen discipline. Switzerland has 45 different federal specialist titles. In addition to these, other qualifications can be obtained as additional competences or sub-specialisations by means of sub-specialties and proficiency certificates. There are currently 39 monodisciplinary and eight interdisciplinary sub-specialties and 42 proficiency certificates. Monodisciplinary sub-specialties correspond to a sub-specialisation in an individual specialist area. Interdisciplinary sub-specialties and proficiency certificates are deemed confirmation that the holder has completed postgraduate training or continuing education which does not satisfy the requirements of a specialist title in terms of scope or importance, or for a completed postgraduate training or continuing education in specific investigation or treatment methods (SIME Specialist Training Regulations). The Swiss Institute for Medical Education (SIME) can propose general aspects of postgraduate medical training through changes to the Specialist Training Regulations and to the general learning objectives that are mandatory for all specialist titles. 

#### SIME’s general learning objectives

The SIME’s general learning objectives are an integral part of the Specialist Training Regulations that are mandatory for all Swiss specialist postgraduate training programmes. They are based on the various roles of doctors, described according to CanMEDS [14]. They include competencies in the fields of communication, collaboration, health promotion, economics and medicine, team and conflict management, leadership, dealing with errors, and ethical decision-making. What all these topics have in common is that while they contain knowledge aspects, they are much more about values, attitudes and emotional aspects. They are therefore particularly suited to transformative education approaches, as also called for by initiatives such as the German Climate and Health Alliance [https://www.klimawandel-gesundheit.de/]. Central elements of transformative learning are interactive events, the sharing of experience within occupational groups, as well as interdisciplinary and interprofessional programmes. Health professionals should also benefit in particular from the expertise of representatives of climate and environmental research. 

Planetary health is to be included as a new topic in the general learning objectives. This will place the onus on the medical specialty societies to incorporate aspects of planetary health as an overarching topic in their specialist fields in the learning content of postgraduate training and continuing medical education. At a later stage of implementation of the planetary health strategy, the development of an interdisciplinary sub-specialty or a proficiency certificate in planetary health is a possibility. The potential learning content for this is set out in table 2 (Tab. 2). 

#### 2.2. Planetary health in continuing medical education

Lifelong continuing education starts after completion of postgraduate training and is essential to allow practitioners to stay abreast of the latest developments in their fields and keep their knowledge and skills up to date. Here, too, aspects of planetary health could be incorporated, by calling on the medical specialty societies to include the topic areas set out in table 2 (Tab. 2) in their curricula. The Swiss Institute for Medical Education (SIME) itself can offer or accredit non-specialised events in overarching fields, such as medical ethics, health economics, patient safety, risk and error management, management and leadership, teaching, communication, medical law, emergency services, evidence-based medicine, medical decision-making, and pioneering research and technology. However, it could also incentivise climate-friendly events in the form of continuing education credits. Specific criteria for congresses and continuing education events could therefore be set out and rewarded with additional credits. For example:

- Encouraging participants to use public transport to travel to events, for example by reimbursing a portion of the public transport costs or including a public transport ticket in the participation fee
- Using an app for general congress information and doing without printed programmes
- Dispensing with industry partner brochures
- Offering necessary printouts on recycled paper only
- Not using disposable crockery
- Incorporating local and seasonal products in catering
- Offering vegetarian or vegan food

#### 2.3. SIME project funding with a special focus on education in the field of planetary health

SIME project funding is an instrument to improve postgraduate medical training. Through financial support for selected postgraduate training projects, the Swiss Institute for Medical Education (SIME) seeks to improve postgraduate training and help ease the burden on doctors responsible for postgraduate training programmes. The SIME project funding is aimed at doctors (both individuals and teams) with a postgraduate training role at an accredited Swiss postgraduate training institution. A panel made up of doctors from different specialties and training experts assess the projects submitted with regard to their benefit to postgraduate medical training, breadth of possible uses, transferability and applicability at other institutions. As well as general educational projects, the project funding can also include specific and important topics and therefore focus on relevant initiatives. A call for proposals on the topic of planetary health is planned. 

## 3. Evaluation of the Planetary Health Strategy in postgraduate medical training and continuing medical education

Besides increasing doctors‘ knowledge of planetary health, the Planetary Health Strategy primarily seeks to generate practical resources and guidance that will bring about behavioural change in both professional and non-work settings. At the same time, doctors can serve as role models for their patients and encourage them to change their behaviour. 

When analysing the highlighted interventions, a distinction must be drawn between short-term changes (knowledge gains and smaller changes in day-to-day work) and medium- to long-term impacts. It is also worth bearing in mind that educational interventions can be evaluated relatively easily using questionnaires, self-assessments and focus groups, but the much more interesting long-term changes are often so multi-factorial that it is difficult to draw direct conclusions about educational activities. 

## 4. Next steps

The project on planetary health in postgraduate medical training and continuing medical education is in the development stage. Some initial ideas have been outlined, as set out above. This list of potential areas of action now needs to be expanded and fleshed out with potential measures. The following activities are planned:

- Deploying an expert group on planetary health in postgraduate training and continuing medical education
- Setting up a think tank to look at possible topic areas and activities
- Networking with national and international stakeholders
- Applying the Delphi method to prioritise the measures
- Expanding on the prioritised measures
- Implementing the initiatives outlined above
- Evaluating the activities carried out and getting the expert group to decide on whether to continue them

## 5. Conclusion

The need for action in terms of greenhouse gas reduction, climate protection and promotion of planetary health has become clear in the last few years. Only through comprehensive and workable measures can the current trend be slowed down or ideally stopped altogether so that future generations can continue to enjoy a healthy and intact environment. The FMH and SIME are prepared to play an active part in raising awareness of the health impacts of climate change across all generations in the medical profession, and to take relevant action themselves and support measures. This will require awareness of these processes at all levels, from policymaking, associations and organisations, and universities or training institutions, to surgeries and hospitals. In the interests of sustainability, the FMH and SIME will work to firmly establish the issue of planetary health within postgraduate training and continuing education for the medical profession. Good national networks and transnational collaboration in the medical education and training sector must be sought so that the interventions and activities in the field of planetary health are as diverse, efficient and sustainable as possible. Those joining the medical profession today are the leaders of tomorrow – they will have to grapple with the health impacts of climate change when treating their patients, and they may have some tough decisions to make in future. To ensure the long-term health of all, an interprofessional and interdisciplinary understanding of the seriousness and urgency of the current situation is needed and action must be taken that is tailored to the problem at hand. 

## Competing interests

The authors declare that they have no competing interests. 

## Supplementary Material

Planetary Health – Strategy on the courses of action on climate change fort he medical profession in Switzerland

## Figures and Tables

**Table 1:**
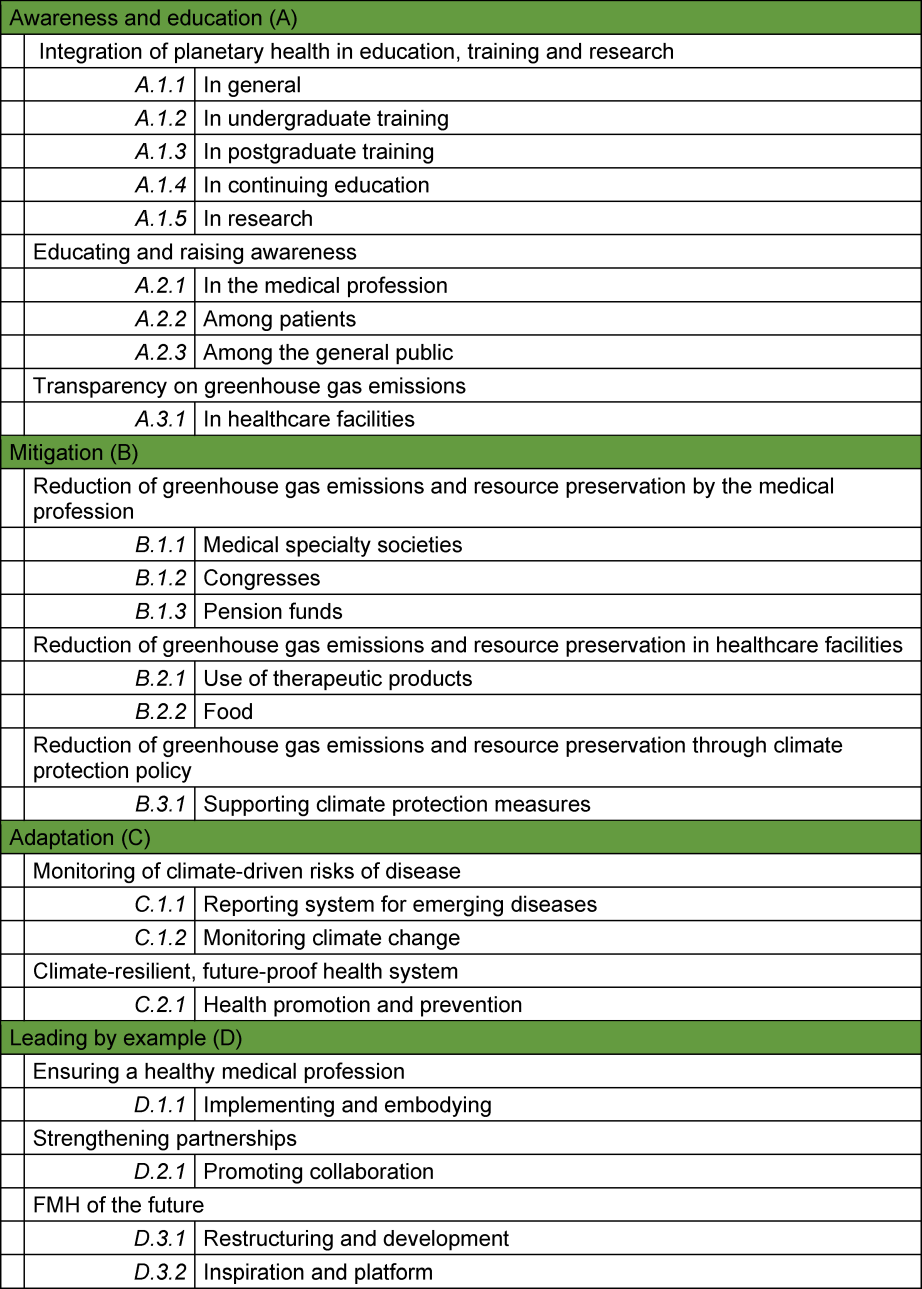
Fields of action and goals of the planetary health strategy

**Table 2:**
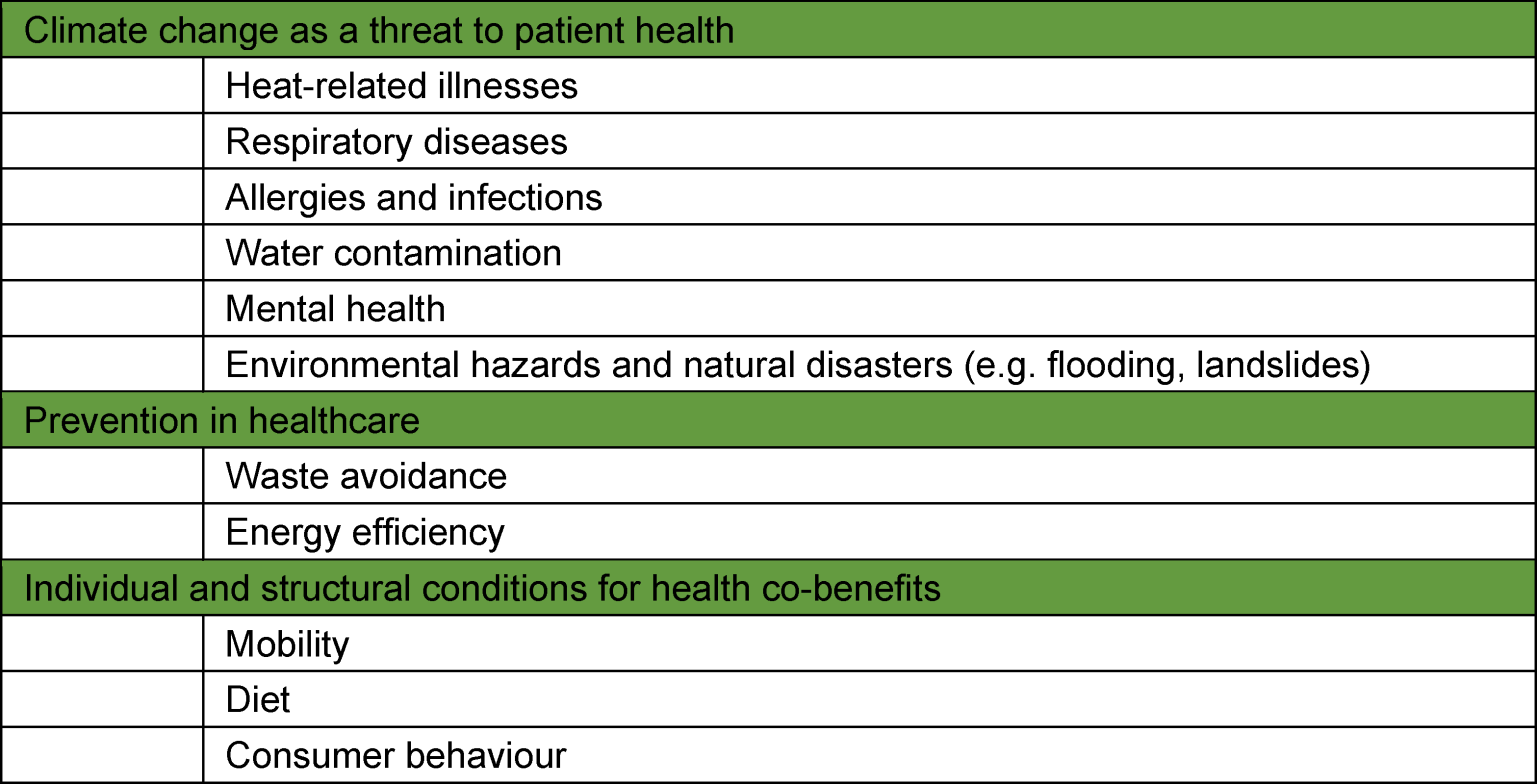
Learning conent for postgraduate training and continuing medical education
