# Supplementary material for: Initiative for the implementation of planetary health in postgraduate medical training and continuing medical education in Switzerland
Source: GMS J Med Educ. 2023 May 15;40(3):Doc26. doi: 10.3205/zma001608 (PMC10291353; doi:10.3205/zma001608)
Supplement: Planetary Health – Strategy on the courses of action on climate change fort he medical profession in Switzerland [file JME-40-26-s-001.pdf]

Attachment 1: Planetary Health – Strategy on the courses of action on climate change fort he medical profession in Switzerland

Attachment 1 to Rieser R, Weil B, Jenni N, Brodmann Maeder M. *Initiative for the implementation of planetary health in postgraduate medical training and continuing medical education in Switzerland.* GMS J Med Educ. 2023;40(3):26. DOI: 10.3205/zma001618

Planetary health - Strategy on the courses of action on climate change for the medical profession in Switzerland

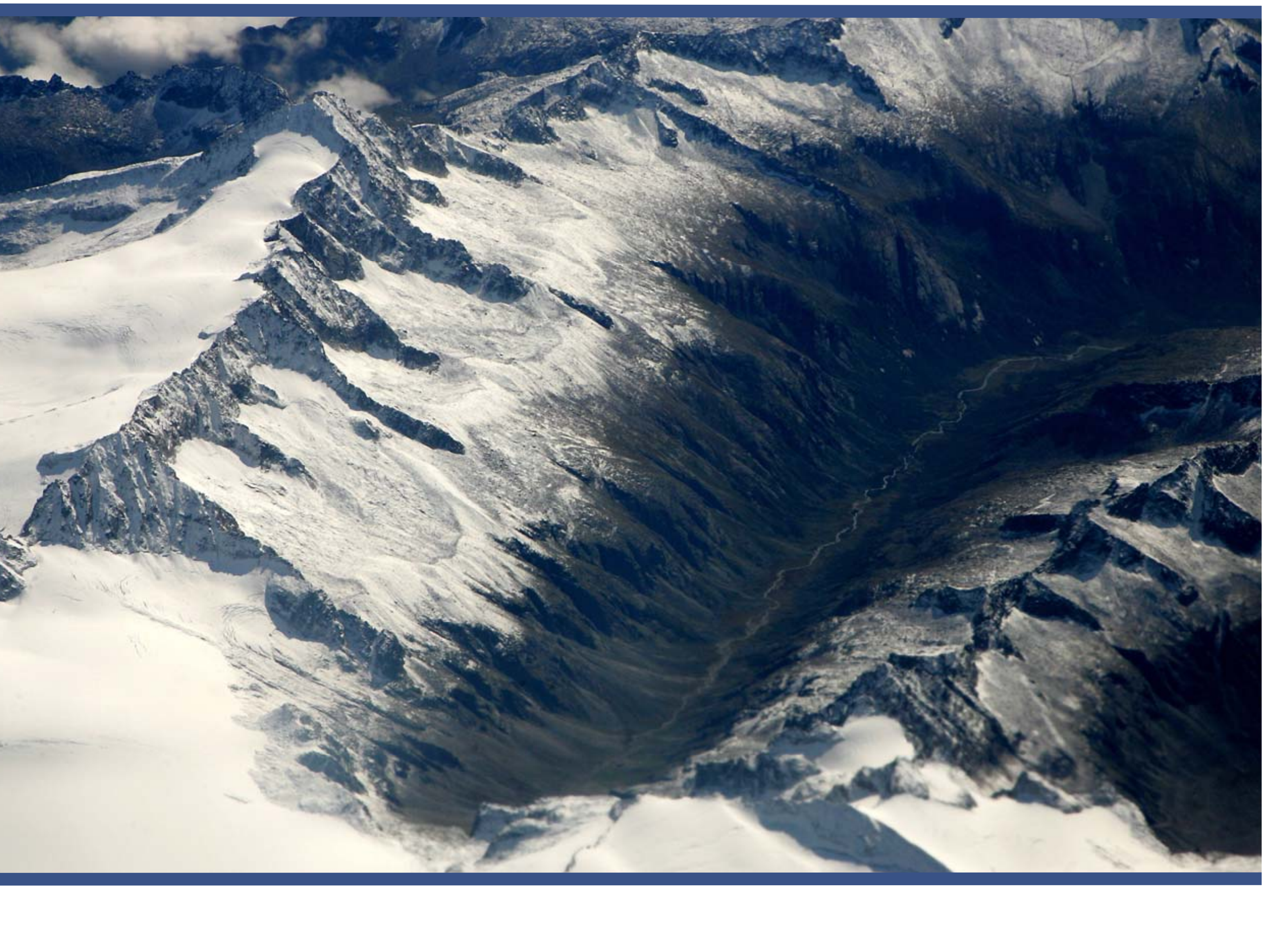

Highest consultation body: Swiss Medical Chamber, Bern, 7 October 2021

Table of contents

1 Starting position **3**
2 Vision **4**
3 Mission & goals **4**

3.1 Awareness and education (A) 5
3.2 Mitigation (B) 7
3.3 Adaptation (C) 8
3.4 Leading by example (D) 9

4 Background information **10**

4.1 Climate change and health 10
4.1.1 Observed evolution of Switzerland’s climate 10
4.1.2 Future evolution of Switzerland’s climate 10
4.1.3 Impact of climate change on health: focus on Switzerland 11

4.2 Climate protection and adapting to climate change: why does climate change particularly affect the medical profession? 14
4.2.1 Climate change poses a risk to health at regional and global level 14
4.2.2 Healthcare sector contributes to domestic greenhouse gas emissions 14
4.2.3 The medical profession helps protect and promote people’s health 14
4.2.4 The medical profession can address the prevention of non-communicable diseases and climate action at the same time 15

5 References **16**

**Working group**	Bea Albermann (swimsa)<br>Franco Denti (OMCT)<br>Philippe Eggimann (SMSR)<br>Michele Genoni (FMCH)<br>François Héritier (KHM)<br>Nadja Jenni (SIWF)<br>Federico Mazzola (vsao)<br>Hans Vogel (VEDAG)
**Scientific support**	Martina Ragettli (Swiss TPH)<br>Martin Röösli (Swiss TPH)
**FMH General Secretariat**	Bruno Henggi (Public Affairs)<br>Ursina Pally (Secretary General)<br>Charlotte Schweizer (Communication)
**Management**	Carlos Quinto (FMH Central Committee – Head)<br>Michel Matter (FMH Central Committee – Deputy Head)
**Coordination**	Robin Rieser (Public Health division)<br>Barbara Weil (Public Health division)

1 Starting position

Climate change is the biggest global health threat of the 21st century.[1] Both the Federal Council's health policy strategy for the period 2020–30[2] and the current COVID-19 pandemic highlight the importance of taking a holistic approach to human health and its complex relationship with the environment.

'Planetary health' deals with the health of human civilisation and the state of the natural systems on which it depends. It therefore describes a concept in which the health of the planet is of central importance as the basis of human health and all life.[3] The concept utilises synergies by taking a holistic approach, for example protecting, preserving and improving the natural environment and also stabilises the climate, protects health and promotes a sustainable economy. In this document, planetary health is understood to be a comprehensive cross-sectoral concept, which incorporates previous and similar multidisciplinary concepts, such as 'GeoHealth', 'EcoHealth' and 'OneHealth'.

It is undisputed that the climate is changing and average temperatures are rising due to greenhouse gas emissions. The sixth assessment report by the Intergovernmental Panel on Climate Change (IPCC) illustrates the extent of anthropogenic climate change. In many areas, the changes that have already occurred are no longer reversible or are inevitable. The world's climate is set to be characterised in the coming years by increasing average global temperatures, rising sea levels, melting glaciers, periods of drought and heavy rain. The changes to the climate will occur sooner than predicted back in 2018, with the 1.5° C rise in average global temperatures to be reached as early as 2030. According to the IPCC, there is an urgent need for action across all sectors to minimise the impact of these changes.[4] Switzerland signed the Paris Agreement in 2015 and ratified it in 2017. At domestic level, implementation of the Paris Agreement requires greenhouse gas emissions to be halved by 2030 compared with the 1990 baseline.

If all the world's health systems were a country, it would be the fifth biggest polluter on Earth. According to the recent Lancet Countdown Report,[5] global healthcare accounts for 4.6% of total emissions. By way of comparison: global aviation accounts for 2–2.5% of all anthropogenic CO_2 emissions globally. In Europe, the German, Swiss and UK health systems are classified as the main emitters in the healthcare sector. Their CO_2 emissions are between 0.66 and 1.02 tonnes per capita.[6] For comparison: civil aviation departing from Switzerland emits around 0.63 tonnes of CO_2 per capita.[7]

Changes to the greenhouse gas emissions from the Swiss health system cannot be achieved through efforts by individual institutions. Cross-sectoral collaboration and action are essential to reduce greenhouse gas emissions.

The FMH Delegates Assembly recognised climate change as a substantial threat to regional and global health on 3 September 2020 and this was duly noted by the Medical Chamber on 29 October 2020. As the occupational group with the highest level of public trust worldwide, doctors have a special responsibility to work to protect and promote health. [8] Through this strategy, the FMH is assuming the special responsibility of the medical profession and together with the umbrella organisations, developing courses of action based on a vision and four missions.

The FMH is guided by the recommendations of the World Health Organization (WHO)[9] and examples from other countries (England, Germany, United States).

2 Vision

“The Swiss medical profession supports strengthening action to promote planetary health.

It has a vision of a Swiss health system that sustainably promotes health and is climate resilient, and it helps achieve this goal through proportionate and affordable measures.”

3 Mission & goals

The climate crisis poses many challenges to human health.

Action is needed in terms of:

- **A.** Raising awareness and educating the medical profession and patients
- **B.** Reducing greenhouse gas emissions (mitigation)
- **C.** Adapting to the foreseeable climatic changes
- **D.** Reinforcing the role of doctors as role models.

For each of these areas, clear goals and measures are set out. Indicators help evaluate measures and adapt them where necessary to deliver on sustainable development goals. For each indicator, there is a date (2023, 2025, 2030) stating when it should be achieved by.

As part of its (professional) policy work and its involvement in drawing up submissions in the area of healthcare, the FMH will consider the stated need for action. The Public Health, Healthcare Professions and Therapeutic Products department will deploy an advisory group, whose members are representative of the member organisations that are entitled to have a say. The advisory group will measure the services and political proposals launched by the FMH or on which FMH has expressed a view in terms of whether they take sufficient account of the stated objectives. The Central Committee reports once a year on the progress of work to the Delegates Assembly and the Medical Chamber.

3.1 Awareness and education (A)

The Swiss medical profession should be aware of its courses of action and work to ensure that knowledge and competencies on planetary health are developed and disseminated. This comprises the following core points:

- the evidence-based interrelationships between the environment, climate change, socioeconomic conditions, lifestyle and health, particularly the health and social effects of climate change;
- the co-benefits of environmental protection and health promotion (mobility, food, connecting with nature etc.);
- climate communication, the medical profession's role in raising awareness of the health effects of climate change, and the courses of action in the area of planetary health, as well as addressing a healthy and sustainable lifestyle drawing on their recognised expertise in doctor-patient consultations.

**Goals**			**23**	**25**	**30**
Integrating planetary health in education and training	… in general	The SIME will develop an overarching strategy on integrating planetary health in medical training based on the recommendations of the Association for Medical Education in Europe AMEE[1]	**X**		
	... in undergraduate medical training	In collaboration with students, the SIME and FMH will use their positions in relevant commissions and bodies (e.g. SMIFK) to champion the Switzerland-wide and sustainable integration of planetary health in the curriculum.	**X**		
	... in postgraduate training	The SIME and FMH will encourage the medical specialty societies to integrate planetary health in their postgraduate training programmes and in their training regulations, and will provide the relevant support to training establishments. The SIME will integrate planetary health as a central topic in the general learning objectives.	**X**		
	… in continuing education	The SIME and FMH will encourage their member organisations to include planetary health as a cross-cutting topic at medical congresses. The SIME will create incentives to accredit relevant events and develop other incentives for climate-neutral congresses and other offerings (climate credits).		**X**	

[1] AMEE Consensus Statement: Planetary health and education for sustainable healthcare

	… in research	The FMH and its members will advocate the expansion of transdisciplinary research and knowledge and support research interests on planetary health and ideally on co-benefits.	X	X	
Educating and raising awareness	… in the medical profession	The FMH will work to make relevant information on planetary health easily accessible and regularly communicate any relevant developments and scope for action to the medical profession.	X		
	… among patients	The FMH will work to make sure that the medical profession provides patients with readily understandable information on climate-friendly and healthy lifestyles and health co-benefits. It will recommend its members incorporate this topic in doctor-patient consultations whenever possible and appropriate.	X		
	… among the general public	The FMH will perform its role as a health advocate and communicate with the public/media and policymakers on issues around health protection in the context of planetary health.	X		
Transparency on greenhouse gas emissions	… in healthcare facilities	The FMH and its member organisations support the recording and publication of emissions in Swiss healthcare facilities.	X		

3.2 Mitigation (B)

In performing its task of delivering healthcare to the public, the Swiss medical profession should play an active part in reducing greenhouse gases and preserving resources. The opportunities this affords should in turn help achieve the federal government's emissions reduction targets.

**Goals**			**23**	**25**	**30**
Reduction of greenhouse gas emissions and resource preservation	… in the medical profession	The FMH will encourage the medical specialty societies to analyse what emissions in their field are composed of and how they can be reduced.	**X**	**X**	
		The FMH and SIME will encourage the medical specialty societies to make congresses climate neutral.[2]	**X**		
		In their pension funds, the FMH and its member organisations will work towards a fundamentally sustainable investment strategy.[3]	**X**		
	... in healthcare facilities	In day-to-day clinical practice, the FMH and its member organisations will work within their powers and competencies to ensure sustainable use of therapeutic products throughout the supply chain.	**X**		
		The medical profession will work to ensure that the food served is sustainably sourced and healthy, in accordance with the current national and international dietary guidance.[4]	**X**		
	... through climate protection policy	The FMH, its member organisations and its members support climate protection measures related to health at all levels wherever possible.	**X**		

[2] https://easac.eu/publications/details/decarbonisation-of-the-health-sector/
[3] https://www.sif.admin.ch/sif/de/home/finanzmarktpolitik/nachhalt_finanzsektor.html
[4] E.g. in line with the Lancet EAT report of 2019

3.3 Adaptation (C)

The Swiss medical profession should work to ensure that Switzerland and its health system adapts and is resilient to a changing climate, with a focus on health promotion and prevention and special protection for vulnerable population groups.

Goals			23	25	30
Monitoring	… of climate-related risks of disease	The FMH supports the establishment of a reporting system for emerging diseases.	X		
		The FMH supports data collection, monitoring and early detection of the health impacts of climate change.	X	X	X
Promoting a climate-resilient, future-proof health system	... through health promotion and prevention	The FMH will work within the scope of public health to ensure that diseases related to planetary health (e.g. zoonotic and vector-borne diseases) are considered at an early stage in epidemic prevention.	X		

3.4 Leading by example (D)

The Swiss medical profession should champion a policy that protects and promotes public and planetary health. It therefore leads by example,[5] by promoting co-benefits, among other things.

Goals			23	25	30
Ensuring the long-term health of the medical profession	… by implementing and embodying a sustainable approach to health	With regard to the relevance of climate targets to health, the medical profession will strive to take a sustainable approach to the design of working environments and will lead by example in this sense.[6]	X		
Strengthening partnerships	… by promoting collaboration with various organisations and sectors	The FMH and its member organisations position themselves in medical specialty societies and bodies to promote a sustainable health system and planetary health. In this area, they will support partnerships, projects, programmes and initiatives.		X	
Building the FMH of the future	… through restructuring and development	The FMH General Secretariat and the SIME administrative office will take the relevant steps to reach net zero in their operations by 2030.	X		
	… through inspiration and platform	Together with other partners in the health sector, the FMH will instigate a Sustainable Health Care Excellence Award to recognise projects, programmes and special achievements by people or institutions in the area of health protection through climate protection.	X		

[5] As per PROFILES: https://www.profilesmed.ch/
[6] As per PROFILES: https://www.profilesmed.ch/

4 Background information

4.1 Climate change and health

4.1.1 Observed evolution of Switzerland's climate

Switzerland's climate has warmed by around 2 °C on average since the mid-19th century. This rise in temperature is around twice as high as the global average temperature increase. Owing to its geographical location (continental position, relative proximity to the warming polar regions) and potential feedback effects (e.g. related to the reduction in Alpine snow cover), Switzerland is particularly affected by global warming. This warming is primarily driven by greenhouse gas emissions caused by human activities. Warming has considerably accelerated since the 1980s. Switzerland's warmest years on record have all been recorded since 2010 (Figure 1).[10] The six warmest years globally have occurred since 2015.[11] Various climatic changes illustrate climate change in Switzerland (Figure 2). Extreme temperatures and heavy precipitation have markedly increased, while snow cover on the Swiss plateau has decreased. The number of heatwaves, very hot days (temperatures in excess of 30 °C) and tropical nights has significantly increased in recent decades. For example, in the 1960s, Ticino would see one to two very hot days a year on average, whereas now that number is 20 or more. A rise in the number of very hot days has also been observed at other measuring stations in Zurich and Genev[GB1]a.[10]

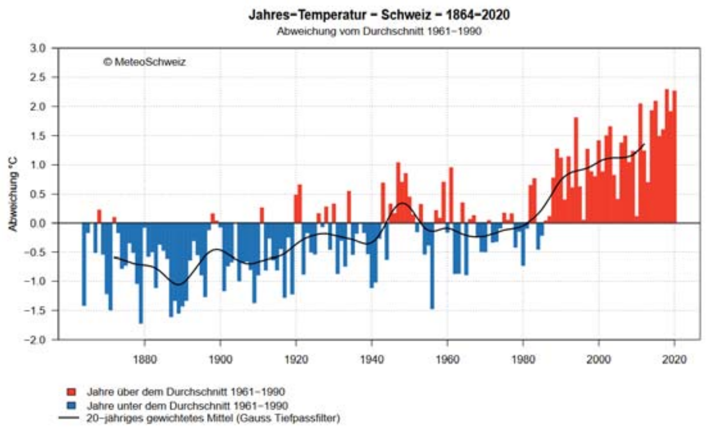

Figure 2: Average annual temperature in Switzerland from 1864 to 2020. The chart shows the deviations from the annual values and the average of the period 1961–90. Source: MeteoSwiss

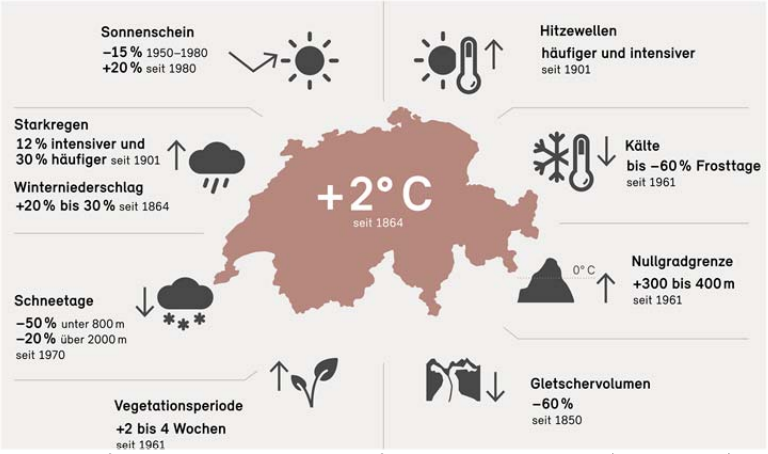

Figure 1: Observed changes in the Swiss climate to date (as at 2019). Source: FOEN, MeteoSwiss, NCCS[6]

4.1.2 Future evolution of Switzerland's climate

The Swiss climate scenarios CH2018 assume that Switzerland is heading for a further 2 °C to 3 °C of warming by the middle of this century if no comprehensive global climate action is taken. According to this scenario, the frequency and intensity of heatwaves is set to further increase. By 2060 average summer rainfall will also decrease by up to 25% and heavy precipitation will occur 10% more often. A rapid and comprehensive reduction of global greenhouse gas emissions would allow the additional warming of the Swiss climate to be limited to an average of +0.7 °C to +1.9 °C by the middle of this century. Through systematic climate action, two thirds of the potential impact on Switzerland's climate could be avoided by 2060, and the goals of the Paris Agreement signed in 2015 (of limiting global warming to well below 2 °C compared with pre-industrial levels) would likely be achieved. At domestic level, implementation of the Paris Agreement by 2030 requires greenhouse gas emissions to be halved compared with 1990 levels.[10]

4.1.3 Impact of climate change on health: focus on Switzerland

The changes to Switzerland's climate have implications for human health. Changes in the climate and weather can have a direct and indirect impact on health by altering ecosystems (Figure 3).

Direct effects on health: heat stress and extreme events

These impacts usually occur in the context of extreme heat.[GB2] In Switzerland, the increasing heat stress was classified by the Federal Office for the Environment (FOEN) as a priority climate-related risk.[12] High temperatures put strain on the human body. Heat can cause exhaustion and heatstroke, and make existing conditions worse, such as cardiovascular diseases, respiratory diseases, kidney disease or mental disorders.[5] Rising temperatures also increase the risk of infections and wound healing disorders. [13] Heatwaves and individual hot days caused both an increase in emergency hospital admissions[14] and mortality in Switzerland. [15,16] Studies show that from maximum daily temperatures of 30 °C, the heat-related mortality risk is significant and rises sharply with every 1 °C increase. Tropical nights (when the temperature does not drop below 20 °C) are an additional health risk as the body does not get a chance to rest and recover at night if the temperatures do not drop. [17] The increasing heat stress can also affect people's wellbeing. High temperatures have an adverse impact on human performance, and on productivity and concentration in the workplace. [5]

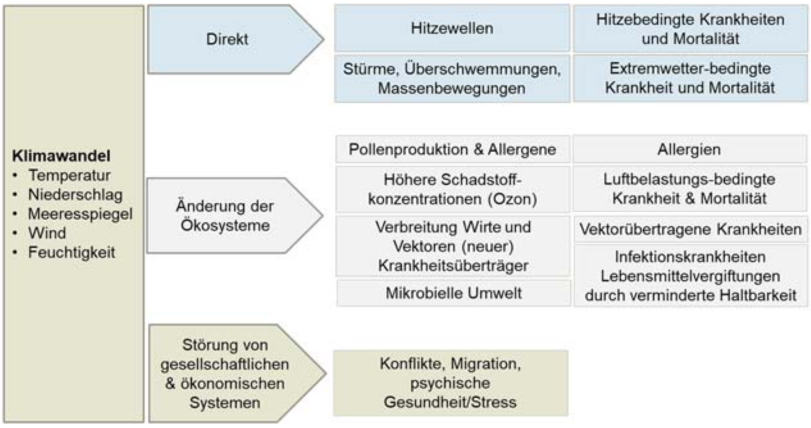

Figure 3: Direct and indirect impacts of climate change on health. Source: Swiss TPH, adapted from [21, 22, 25]

Studies also suggest that high temperatures have a negative impact on mental health. Research in the United States and in Australia has shown that heatwaves stir aggressive behaviour and cause crime rates to rise. A number of recent international studies also point to an increase in suicides in the warmer spring and summer months. Scientific evidence on the causes of such mental health effects and potential influences of other (social) factors are currently scarce, but are attracting increasing interest.[18–20]

High temperatures in the four warmest summers to date (2003, 2015, 2018 and 2019) also caused excess deaths in Switzerland (see table below).

Summer	Ranking of hottest summers	Excess deaths (number)	Excess mortality (%)
2003	1	975	6.9

2015	2	804	5.4
2018	4	185	1.2[a]
2019	3	521	3.5

[a] not statistically significant. In 2018, excess mortality was limited to the month of August (+3.4%).
Source: Swiss TPH [15]

The record-breaking summer of 2003 saw the highest heat-related excess mortality to date. Between July and August that year, around 1,000 more people than usual died, which equates to excess mortality of 6.9%.[21] And in the second-hottest summer in 2015, the impact on mortality was significant, with some 800 excess deaths. [16] People aged 75 or over are the worst affected. In the summers of 2018 and 2019, excess mortality was slightly lower than in previous heatwaves. It may be that the measures put in place by cantonal health authorities and public awareness of heat-related health risks helped protect people. This highlights the importance of such preventive measures, especially given the growing number of very hot days and heatwaves.[15]

The potential increase in personal injuries related to mass wasting (such as rockslides, rockfalls and landslides), storms and flooding is also treated as a priority climate-related risk in Switzerland. This assessment was carried out in 2017 by the FOEN based on a potential accumulation of such events in connection with climate change.[12]

Indirect effects through changing ecosystems and higher concentrations of air pollutants

Health risks related to changing ecosystems are brought about by the potential spread of disease carriers (vectors), pathogens and allergenic plants. Infectious diseases that are food-, water- and vector-borne could become more common. The most important infectious disease vectors in Switzerland are mosquitoes and ticks.

Warmer winters favour the survival of the Asian tiger mosquito (Aedes albopictus) – a potential carrier of the Chikungunya, Dengue and Zika viruses. In recent years, isolated populations of tiger mosquitoes have been detected in Ticino and also north of the Alps. [10] This increases the risk of autochthonous transmission of diseases in Switzerland, like e.g. in Rome and Calabria in 2017 (239 cases of Chikungunya virus) and in Var, France (17 cases of Chikungunya) (Figure 4).[22] The first death from autochthonous malaria involving a four-year-old girl in Northern Italy also made headlines in 2017.[23] Climate change could also favour other pathogens, such as the West Nile virus, that can be spread by native species of mosquitoes.

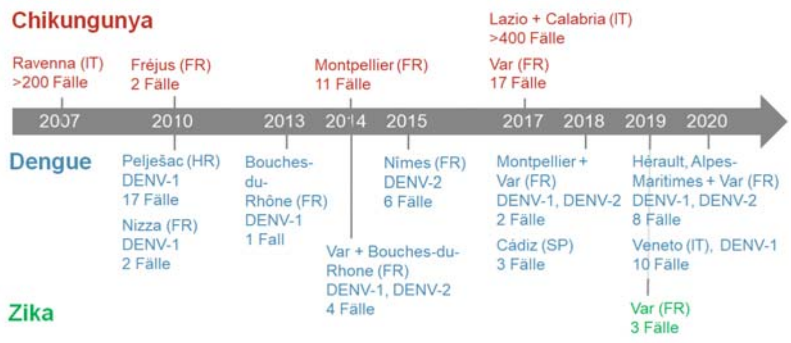

Figure 4: Local disease outbreaks in continental Europe in connection with the Asian tiger mosquito. Source: Swiss TPH

Ticks spread at higher altitudes and an extension of their seasonal activity has been observed. The warmer weather and associated change in leisure activity and clothing also increase the likelihood of coming into contact with ticks. Lyme disease and early summer meningoencephalitis (TBE) are the most common diseases transmitted by ticks in Switzerland. There has been a sharp rise in cases of TBE in Switzerland since 2008 (Figure 5).

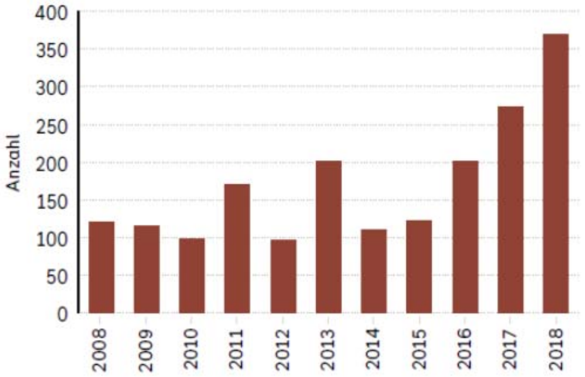

Figure 5: Number of TBE cases per year in Switzerland. Source: FOPH[6]

Climate change will lead to a prolonged pollen season in certain plant species. MeteoSwiss has already noted that the grass pollen season is starting earlier. A higher pollen count and a longer pollen season could increase allergic reactions and lead to a rise in the number of people in Switzerland with allergies.[10]

In addition, future climatic conditions could favour waterborne and foodborne infectious diseases. The production and storage life of foodstuffs are connected in many ways to climatic conditions. Food hygiene is set to become more important, particularly on hot days. The risk of bacterial contamination of drinking water due to changed water temperatures has to date been classified as low due to regular quality checks.[12]

Climate-related long-term changes in weather also favour the spread and secondary formation of air pollutants such as ozone and particulate matter, and therefore have an indirect impact on respiratory and cardiovascular morbidity and mortality.[24] In 2019, air pollution caused over 6.5 premature deaths worldwide, which makes it one of the most important environmental risks to health. The European Environment Agency estimates that there were 3,500 premature deaths in Switzerland in 2018 due to particulate matter and 350 due to ozone. Additional health impacts of short-term increased levels of ozone pollution, for example, include emergency consultations due to diseases of the respiratory tract (e.g. asthma and chronic obstructive pulmonary disease (COPD)) and the accompanied work absences, as well as a measurable deterioration of symptoms or state of health in both healthy people and those who were previously sick.[25]

The anticipated rise in temperatures will lead to longer periods with high ozone concentrations and will also cause the maximum ozone concentrations to increase. Heatwaves particularly favour ozone build-up at ground level. Stable high pressure systems, which favour high concentrations of pollutants, will also occur more frequently.[12,26]

Disruption to social and economic systems

The global changes in environmental conditions triggered by climate change, including rising sea levels and biodiversity loss may – together with a host of other (climate-independent) causes – lead to social conflicts, stagnating development in affected areas, resettlement, migration and thus increased social instability. This may affect both the physical and mental health and emotional wellbeing of populations and of migrants. Natural disasters – such as a cyclone or prolonged drought – can impact the local economy and infrastructure for years. It is well known that those affected by extreme events and resettlement are more likely to suffer from depression, post-traumatic stress disorder, addictions, anxiety and suicidal ideation. People with mental ill health tend to be more affected than those who are mentally healthy.[12,24,27]

In a globalised world, such events abroad can also result in risks in Switzerland. For example, an extreme event abroad could impact the production and transport of medical equipment overseas and therefore restrict the availability of medicinal products in Switzerland.[28]

Even discussing the topic of climate change and the potential events and threats it can cause can affect mental health. Climate change is a chronic environmental stressor that is constantly there and ever-present in the media. [24] Studies from Australia and the United States have shown that the topic can trigger anxiety and depressive symptoms.[29]

4.2 Climate protection and adapting to climate change: why does climate change particularly affect the medical profession?

4.2.1 Climate change poses a risk to health at regional and global level

Climate change has an impact on physical and mental health.[5,30] In many places, there is a danger that the gains of the past in terms of global health will be compromised by global warming. We can assume that in future, certain diseases will occur more often (e.g. TBE, heat-related diseases, pollen allergies) and that others (e.g. vector-borne infectious diseases) will re-emerge. The efficacy, side-effect profile and toxicity of certain medicinal products changes at high temperatures. For example, blood pressure-lowering drugs (beta blockers) reduce the body's ability to adapt to the heat, while neuroleptics and antidepressants can cause a side effect of increasing the body temperature, which is amplified by heat-waves. The future health of society therefore depends on how it deals with climate change.

4.2.2 Healthcare sector contributes to domestic greenhouse gas emissions

The healthcare sector makes a significant contribution to domestic greenhouse gas emissions. Internationally, the healthcare sectors accounts for 4.6% of total emissions according to the latest Lancet Countdown report. [5] In the United States, for example, the level is particularly high, with the domestic healthcare sector accounting for between 8% and 10% of total CO_2 emissions. [31,32] In the United Kingdom, that figure was between 4% and 5% in 2017. Owing to the efforts of the NHS[33] to become the world's first net zero healthcare system, this share continues to fall.[5] The figures for Switzerland differ, depending on the study and calculation method. An international comparison of the carbon footprints of healthcare sectors by Pichler et al.[31] assumed a share of 5.9% for Switzerland in 2014. Meanwhile, Health Care without Harm estimated this figure at 6.7% for 2019.[GB3][6] There are not yet any figures from Swiss studies.

When comparing the domestic greenhouse gas emissions of the healthcare sector, we need to consider the scope of services offered and services used, as well as the greenhouse gas emissions resulting from these services. The latest report of the Lancet Countdown group indicates that high quality of healthcare can still be achieved with lower emissions. A comparison of the Healthcare Access and Quality Index HAQ with the domestic per-capita emissions of the healthcare sector shows that among industrialised nations, there are significant differences in emissions for similar HAQ levels (Figure 6). [5] The healthcare sector therefore has considerable potential as well as a responsibility to contribute to climate protection. This is in line with the goals of the Paris Agreement.

The areas of the healthcare sector with the highest CO_2 emissions are the operation of public and private hospitals, the associated logistics, and the manufacture and transport of pharmaceutical products. The main ways in which the greenhouse gas emissions of the healthcare sector can be reduced are by using less energy and using green energy resources, making buildings sustainable, avoiding waste (including plastics and disposable products), promoting more sustainable production of medicinal products and improving transportation. Promoting telemedicine and avoiding unnecessary treatments and patient transport also reduce greenhouse gas emissions.[31,34]

4.2.3 The medical profession helps protect and promote people's health

Doctors have a special responsibility when it comes to protecting and promoting people's health. This particularly concerns the direct and indirect effects of climate change. The medical profession therefore has an important role to play in communicating, preventing and delivering after-care for the impact of climate change. This applies to their direct dealings with patients, as well as with the general public and

their actions at political level. They can utilise their social status and competencies to help educate the public and policymakers about the connections between climate change, the environment and health.

Underprivileged sections of society are the worst affected by the health impact of climate change. However, conventional preventive measures often fail to adequately reach them. The medical profession (e.g. GPs) has direct access to such population groups, which they can utilise for primary prevention.

in addition, as an occupational group, doctors enjoy a high level of trust among the public and are seen as role models in society. This means that they also have a responsibility to lead by example in their professional dealings.[8,9]

4.2.4 The medical profession can address the prevention of non-communicable diseases and climate action at the same time

Many climate protection measures entail considerable health benefits (co-benefits) as they help preserve the environment and promote health. From the perspective of this dual benefit of climate protection measures, climate change is also considered the biggest opportunity for global health this century.[24] The promotion of sustainable mobility reduces CO_2 emissions and guarantees healthy air quality. At the same time, cycling and walking contribute to physical activity. Cleaner air and more exercise can reduce conditions such as diabetes, cardiovascular diseases and chronic respiratory diseases. This would also bring health cost savings. Food choices also help prevent these kinds of non-communicable chronic diseases and protect the environment.[30] Such relationships should be considered, taking account of the medical and social aspects in consultations between doctor and patient. According to the World Organization of Family Doctors (WONCA), other areas of action with co-benefits for health and the environment are energy choices, reducing environmental impact, connecting to nature and getting involving in the community. The latter is seen as a driver for bringing about changes to a more sustainable and fair society, as well as supporting mental health.[35]

The 2020 Lancet Countdown report talks about a triple win with regard to tackling the COVID-19 pandemic: “Aligning the global COVID-19 recovery with our response to climate change offers the chance to protect health, promote a sustainable economy, and preserve our planet.” [5] In other words, measures to protect health and to deal with the coronavirus crisis benefit the environment, health and the economy. Careful consideration must be given to synergy effects.

5 References

1. Costello A, Abbas M, Allen A, et al. Managing the health effects of climate change: lancet and University College London Institute for Global Health Commission. *The Lancet*. 2009;373(9676):1693-1733.

2. BAG. Die gesundheitspolitische Strategie des Bundesrates 2020–2030. Published 2019. Accessed January 30, 2021. www.gesundheit2030.ch

3. Whitmee S, Haines A, Beyrer C, et al. Safeguarding human health in the Anthropocene epoch: report of The Rockefeller Foundation–Lancet Commission on planetary health. *The Lancet*. 2015;386(10007):1973-2028.

4. IPCC. *Climate Change 2021: The Physical Science Basis. Contribution of Working Group I to the Sixth Assessment Report of the Intergovernmental Panel on Climate Change [Masson-Delmotte, V., P. Zhai, A. Pirani, S. L. Connors, C. Péan, S. Berger, N. Caud, Y. Chen, L. Goldfarb, M. I. Gomis, M. Huang, K. Leitzell, E. Lonnoy, J. B. R. Matthews, T. K. Maycock, T. Waterfield, O. Yelekçi, R. Yu and B. Zhou (Eds.)].* Cambridge University Press. In Press; 2021.

5. Watts N, Amann M, Arnell N, et al. The 2020 report of The Lancet Countdown on health and climate change: responding to converging crises. *The Lancet*. Published online 2020.

6. Health Care Without Harm US & Canada. Health care's climate footprint. How the health sector contributes to the global climate crisis and opportunities for action. Published 2019. Accessed January 31, 2021. https://noharm-uscanada.org/ClimateFootprintReport

7. Bundesamt für Zivilluftfahrt BAZL. CO2-Emissionen des Luftverkehrs. Grundsätzliches und Zahlen. Published online January 2020.

8. Ipsos MORI. Veracity Index 2019. Trust in professions survey. Published 2020. Accessed January 30, 2021. https://www.ipsos.com/sites/default/files/ct/news/documents/2020-11/veracity-index-2020_v2_public.pdf

9. WHO. Did you know: by taking action on climate change you can strengthen public health. Key facts. Published 2015. Accessed January 12, 2021. https://www.who.int/globalchange/publications/didyouknow-health-professionals.pdf?ua=1

10. BAFU, MeteoSchweiz, NCCS. *Klimawandel in Der Schweiz. Indikatoren Zu Ursachen, Auswirkungen, Massnahmen*.; 2020:108.

11. World Meteorological Organization (WMO). 2020 closes a decade of exceptional heat. Medienmitteilung 24. Dezember 2020. Published 2020. Accessed January 11, 2021. https://public.wmo.int/en/media/news/2020-closes-decade-of-exceptional-heat

12. BAFU. *Klimabedingte Risiken Und Chancen. Eine Schweizweite Synthese*. Bundesamt für Umwelt (BAFU); 2017:148.

13. Aghdassi SJS, Schwab F, Hoffmann P, Gastmeier P. The Association of Climatic Factors with Rates of Surgical Site Infections. *Deutsches Aerzteblatt International*. 2019;116.

14. Ragettli MS, Vicedo-Cabrera AM, Flückiger B, Röösli M. Impact of the warm summer 2015 on emergency hospital admissions in Switzerland. *Environmental Health*. 2019;18(66):1-10.

15. Ragettli MS, Röösli M. *Gesundheitliche Auswirkungen von Hitze in Der Schweiz Und Die Bedeutung von Präventionsmassnahmen. Hitzebedingte Todesfälle Im Hitzesommer 2019 – Und Ein Vergleich Mit Den Hitzesommer 2003, 2015 Und 2018*. Schweizerischen Tropen- und Public Health-Institut (Swiss TPH); 2020. https://www.nccs.admin.ch/dam/nccs/de/dokumente/website/sektoren/gesundheit/bericht-gesundheitliche-auswirkungen-von-hitze-in-der-schweiz-und-die-bedeutung-von-praeventionsmassnahmen-korrigier.pdf.download.pdf/SwissTPH_2020_Gesundheitliche%20Auswirkungen%20von%20Hitze_2019_Vergleich%202003-2015-2018_def_corrected.pdf

16. Vicedo-Cabrera AM, Ragettli MS, Schindler C, Röösli M. Excess mortality during the warm summer of 2015 in Switzerland. *Swiss Medical Weekly*. 2016;146:w14379-w14379.

17. Ragettli MS, Vicedo-Cabrera AM, Schindler C, Röösli M. Exploring the association between heat and mortality in Switzerland between 1995 and 2013. *Environmental Research*. 2017;158C:703-709.

18. Palinkas LA, Wong M. Global climate change and mental health. *Current Opinion in Psychology*. 2020;32:12-16. doi:10.1016/j.copsyc.2019.06.023

19. Yu J, Yang D, Kim Y, et al. Seasonality of suicide: a multi-country multi-community observational study. *Epidemiol Psychiatr Sci*. 2020;29:e163. doi:10.1017/S2045796020000748

20. Kim Y, Kim H, Gasparrini A, et al. Suicide and Ambient Temperature: A Multi-Country Multi-City Study. *Environ Health Perspect*. 2019;127(11):117007. doi:10.1289/EHP4898

21. Grize L, Huss A, Thommen O, Schindler C, Braun-Fabrlander C. Heat wave 2003 and mortality in Switzerland. *Swiss Medical Weekly*. 2005;135(13-14):200-205.

22. Gossner CM, Ducheyne E, Schaffner F. Increased risk for autochthonous vector-borne infections transmitted by Aedes albopictus in continental Europe. *Euro Surveill*. 2018;23(24):1800268.

23. Day M. Autopsy results confirm 4 year old Italian girl died from malaria. Published online 2017.

24. Watts N, Adger WN, Agnolucci P, et al. Health and climate change: policy responses to protect public health. *The Lancet*. 2015;386(10006):1861-1914.

25. European Environment Agency. *Air Quality in Europe: 2020 Report.* Publications Office; 2020. Accessed May 10, 2021. https://data.europa.eu/doi/10.2800/786656

26. SwissTPH. LUDOK - Interaktive Grafik zu den Auswirkungen der Luftverschmutzung auf die Gesundheit. Swiss TPH. Published 2020. Accessed May 12, 2021. https://www.swisstph.ch/de/projects/ludok/healtheffects/

27. Smith KR, Woodward A, Campbell-Lendrum D, et al. 2014: Human health: impacts, adaptation, and co-benefits. In: Field CB, Barros VR, Dokken DJ, et al., eds. *Climate Change 2014: Impacts, Adaptation, and Vulnerability. Part A: Global and Sectoral Aspects. Contribution of Working Group II to the Fifth Assessment Report of the Intergovernmental Panel on Climate Change*. Cambridge University Press; 2014:709-754.

28. BAFU. *Auswirkungen Des Klimawandels Im Ausland – Risiken Und Chancen Für Die Schweiz. Zusammenfassung.*; 2020:17 S.

29. Bunz M, Mücke H-G. Klimawandel–physische und psychische Folgen. *Bundesgesundheitsblatt-Gesundheitsforschung-Gesundheitsschutz*. 2017;60(6):632-639.

30. Haines A, Ebi K. The imperative for climate action to protect health. *New England Journal of Medicine*. 2019;380(3):263-273.

31. Pichler P-P, Jaccard IS, Weisz U, Weisz H. International comparison of health care carbon footprints. *Environmental Research Letters*. 2019;14(6):064004.

32. Nikendei C, Bugaj TJ, Nikendei F, Kühl SJ, Kühl M. Klimawandel: Ursachen, Folgen, Lösungsansätze und Implikationen für das Gesundheitswesen. *Zeitschrift für Evidenz, Fortbildung und Qualität im Gesundheitswesen*. Published online 2020.

33. NHS England NI. Delivering a 'Net Zero' National Health Service. Published online 2020. Accessed July 14, 2021. https://www.england.nhs.uk/greenernhs/publication/delivering-a-net-zero-national-health-service/

34. Lenzen M, Malik A, Li M, et al. The environmental footprint of health care: a global assessment. *The Lancet Planetary Health*. 2020;4(7):e271-e279.

35. Senn N. Co-Benefits von Gesundheit und Umwelt. *Schweizerische Akademie der Medizinischen Wissenschaften (SAMW) Bulletin*. 2020;4/2020:2-3.
